# A167 ASSESSMENT OF QUALITY OF LIFE AMONG CHILDREN WITH CELIAC DISEASE IN JORDAN

**DOI:** 10.1093/jcag/gwad061.167

**Published:** 2024-02-14

**Authors:** E Altamimi, L Haj-Ahmad, A Alqaisi

**Affiliations:** Pediatrics and Neonatology, Jordan University of Science and Technology, Irbid, Jordan; The University of Jordan, Amman, Amman, Jordan; Celiac Disease Care Provider Society, Amman, Jordan

## Abstract

**Background:**

Celiac disease has a profound impact on the daily lives of patients who are diagnosed with this chronic disease and affected by its myriad of clinical implications. Thus, health-related quality of life (HrQol) surveys proved invaluable in assessing children with this condition.

**Aims:**

We aimed to assess HrQol of children with celiac disease in Jordan. In addition to the effect of gender, diet-restriction compliance compliance and the concomitant occurance of other chronic illnesses on the HrQol in our cohort.

**Methods:**

A cross-sectional study of all children registered with the Celiac Disease Care Providers Society (CDPS) were asked to fill an online quality of life questionnaire. The Arabic version of the Kidscreen-52 questionnaire, with 10 domains was adopted. Data was analyzed using descriptive statistics and average T-scores across 10 health domains. Data was compared to previously published international norms.The sample was divided into four cohorts based on concomitant disorders, disease duration, adherence to gluten free diet (GFD), and growth issues. Independent sample t-tests, p-values, and Cohen's ds were determined for each cohort. Subcohorts were compared to each other.

**Results:**

Out of 400 children registered with the society. 126(31.5%) patients responded. Male celiac patients performed worse in six health domains compared to the general population: moods and emotions, self-perception, bullying, psychological well-being, social support, and financial resources. The last three of which showed poorer performance in female celiacs too. Moods and emotions and self-perception were significantly worse in males with chronic disease and without GFD adherence. Females with growth issues performed worse in school environment and financial resources.

**Conclusions:**

The study reveals a significant reduction in Hrqol in celiac patients compared to healthy individuals, a first in Jordan. This highlights the need for education initiatives for patients and physicians, as well as campaigns for national support for GFD. Further research is needed to identify other contributing factors and what interventions at community level should be taken.

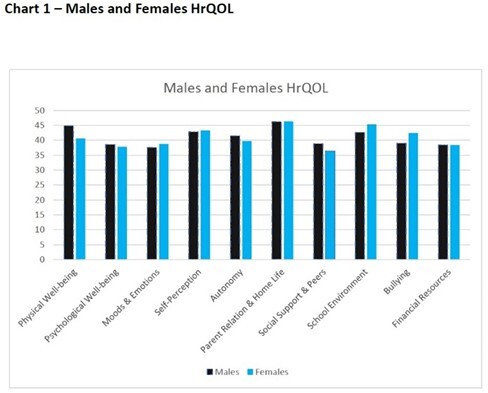

**Funding Agencies:**

None

